# Comparison of Biochemical Properties of HIV-1 and HIV-2 Capsid Proteins

**DOI:** 10.3389/fmicb.2017.01082

**Published:** 2017-06-13

**Authors:** Yasuyuki Miyazaki, Ariko Miyake, Noya Doi, Takaaki Koma, Tsuneo Uchiyama, Akio Adachi, Masako Nomaguchi

**Affiliations:** ^1^Department of Microbiology and Cell Biology, Tokyo Metropolitan Institute of Medical ScienceTokyo, Japan; ^2^Laboratory of Molecular Immunology and Infectious Disease, Joint Faculty of Veterinary Medicine, Yamaguchi UniversityYamaguchi, Japan; ^3^Department of Microbiology, Tokushima University Graduate School of Medical SciencesTokushima, Japan

**Keywords:** HIV-1, HIV-2, Gag-CA, NTD, CA-polymerization, CA-stability

## Abstract

Timely disassembly of viral core composed of self-assembled capsid (CA) in infected host cells is crucial for retroviral replication. Extensive *in vitro* studies to date on the self-assembly/disassembly mechanism of human immunodeficiency virus type 1 (HIV-1) CA have revealed its core structure and amino acid residues essential for CA–CA intermolecular interaction. However, little is known about *in vitro* properties of HIV-2 CA. In this study, we comparatively analyzed the polymerization properties of bacterially expressed HIV-1 and HIV-2 CA proteins. Interestingly, a much higher concentration of NaCl was required for HIV-2 CA to self-assemble than that for HIV-1 CA, but once the polymerization started, the reaction proceeded more rapidly than that observed for HIV-1 CA. Analysis of a chimeric protein revealed that N-terminal domain (NTD) is responsible for this unique property of HIV-2 CA. To further study the molecular basis for different *in vitro* properties of HIV-1 and HIV-2 CA proteins, we determined thermal stabilities of HIV-1 and HIV-2 CA NTD proteins at several NaCl concentrations by fluorescent-based thermal shift assays. Experimental data obtained showed that HIV-2 CA NTD was structurally more stable than HIV-1 CA NTD. Taken together, our results imply that distinct *in vitro* polymerization abilities of the two CA proteins are related to their structural instability/stability, which is one of the decisive factors for viral replication potential. In addition, our assay system described here may be potentially useful for searching for anti-CA antivirals against HIV-1 and HIV-2.

## Introduction

Highly ordered core structure of human immunodeficiency virus type 1 (HIV-1) consisting of multimeric capsid (CA) proteins is essential for modulating the complex virus replication (Freed and Martin, 2013; Goff, 2013). While unusual stabilization by mutations in CA abrogates viral infectivity through incomplete reverse transcription of viral genome (Forshey et al., 2002), rhesus α-isoform of tripartite motif-containing protein 5 (TRIM5α) eliminates viral infectivity by abnormally promoting disassembly of CA proteins (Forshey et al., 2005; Sebastian and Luban, 2005; Stremlau et al., 2006). A variety of host proteins have been reported to regulate CA disassembly: cyclophilin A (CypA) (Braaten et al., 1996a,b; Gamble et al., 1996), PDZ domain-containing protein 8 (PDZD8) (Henning et al., 2010; Guth and Sodroski, 2014), cleavage and polyadenylation specificity factor (CPSF) (Lee et al., 2010; Price et al., 2012), and myxovirus resistance protein 2 (MX2) (Goujon et al., 2013; Kane et al., 2013; Liu et al., 2013). Thus, proper disassembly of the CA-core structure in the viral replication cycle in concert with cellular proteins is critical for HIV-1 infectivity. Given that the process of HIV-1 CA core dissociation in infected cells is intricately mediated by numerous viral and cellular factors, *in vitro* model systems that mimic the *in vivo* situation to a certain extent are required to gain definite insights into molecular events in HIV-1 core formation/deformation. In fact, various *in vitro* systems have been developed to study the physicochemical aspects of HIV-1 CA–CA interaction (Ehrlich et al., 1992; Campbell and Vogt, 1995; Gross et al., 1997; von Schwedler et al., 1998; Gross et al., 2000; Ehrlich et al., 2001; Lanman et al., 2002; Mateu, 2002; Morikawa et al., 2004; Alfadhli et al., 2005; del Alamo et al., 2005; Lidon-Moya et al., 2005; Chen and Tycko, 2010). Although the above systems are influenced by numerous factors, such as ion strength, temperature, pH, and crowding agents, a high concentration of NaCl has been generally and frequently used to initiate the CA-assembly *in vitro*.

**FIGURE 1:**
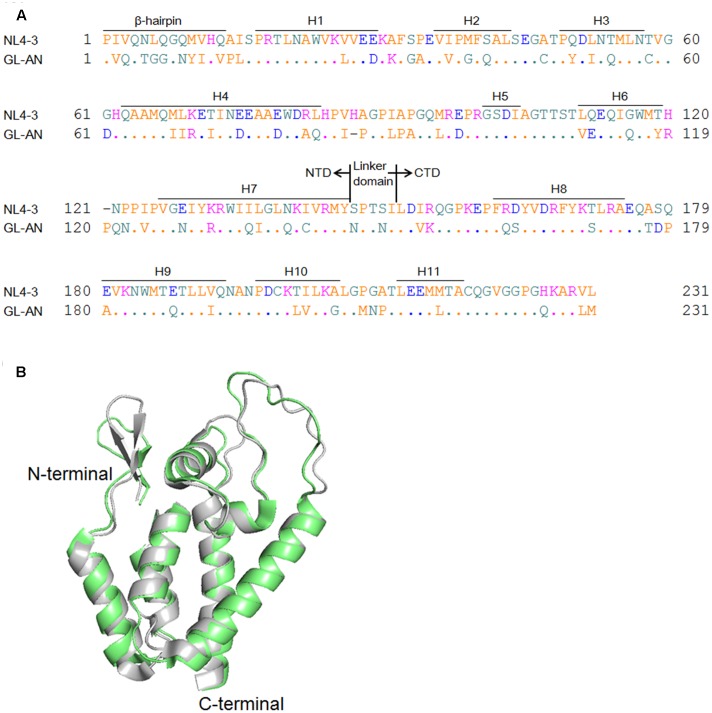
Structural comparison of NL4-3 and GL-AN Gag-CA proteins. **(A)** Alignment of NL4-3 and GL-AN Gag-CA sequences. CA amino acid sequences of HIV-1 NL4-3 (GenBank accession number: AF324493) and HIV-2 GL-AN (GenBank accession number: M30895) are aligned. The N-terminal domain (NTD), linker domain, C-terminal domain (CTD), β-hairpin, and helices 1 to 11 (H1 to H11) are shown based on previous studies (Gamble et al., 1996; von Schwedler et al., 2003; Robinson et al., 2014; Gres et al., 2015). **(B)** Superimposition of the NTD structures. Superposed structures of HIV-1 NTD (green, PDB code: 3H4E) and HIV-2 NTD (gray, PDB code: 2WLV) were depicted by PyMOL ver 1.8.

HIV CA consists of two globular domains [N-terminal domain (NTD) and C-terminal domain (CTD)], and a linker domain connecting these two domains (**Figure 1A**). While HIV-1 CA NTD has an N-terminal β-hairpin and seven α-helices in the downstream region (Gitti et al., 1996; Momany et al., 1996), its CA CTD has four α-helices (Gamble et al., 1997; Du et al., 2011). The former primarily forms a hexameric structure (also forms a pentameric structure), and the latter interacts with the NTD of adjacent CA molecules (Pornillos et al., 2009, 2011). CTD also forms a CTD-CTD dimer (Ganser-Pornillos et al., 2007; Pornillos et al., 2009). Based on cryo-electron microscopy studies, it has been proposed that HIV-1 cores in mature virions have a mixture of approximately 250 hexameric CA proteins and 12 pentameric CA proteins at upper and lower ends of the conical core (Ganser et al., 1999; Zhao et al., 2013). *In vitro* studies have also shown that HIV-1 CA is assembled to form core-like structure made up of hexameric CA proteins, a structure similar to the core in native virions (Ganser et al., 1999; Byeon et al., 2009; Zhao et al., 2013). Of note, this self-assembly process of HIV-1 CA (monomers, hexamers, and final core-like products consisting of hexamers) can be induced by high ionic strength, and readily monitored by simple turbidity assays *in vitro* (Ehrlich et al., 1992; Li et al., 2000; Ganser-Pornillos et al., 2004; Barklis et al., 2009). Moreover, as described above, CA disassembly process regulated by CA inhibitors/host proteins, such as TRIM5α, CypA, and PDZD8, can be experimentally analyzed *in vitro* as well as *in vivo* (Grattinger et al., 1999; Ternois et al., 2005; Black and Aiken, 2010; Guth and Sodroski, 2014).

Amino acid sequences of HIV-1/HIV-2 CA proteins are significantly related to each other (**Figure 1A**), and more strikingly, their NTD 3-D structures are highly similar (**Figure 1B**). However, although HIV-1 and HIV-2 exhibit distinct biological properties associated with their CA proteins (Freed and Martin, 2013), to the best of our knowledge, *in vitro* properties of HIV-2 CA have been very poorly studied so far. HIV-2 is a medically and socially important retrovirus in addition to HIV-1, and is important for basic virology as well. In this study, we comparatively analyzed the *in vitro* polymerization properties of HIV-1/HIV-2 CA proteins, and also their thermal stability. We found that HIV-1 and HIV-2 CA proteins are remarkably different from each other in these characteristics, and demonstrated that the observed difference is attributable to the NTD of CA proteins. Our results here suggest that the structural instability/stability of CA NTD influences distinct biological properties of HIV-1 and HIV-2.

## Materials and Methods

### Plasmids

Sequences encoding a full-length CA of HIV-1 NL4-3 (Pro1-Leu231 in **Figure 1A**) and its NTD (Pro1-Tyr145 in **Figure 1A**) were PCR-amplified and cloned into pET21 (EMD chemicals, Inc.) using *Nde* I and *Xho* I sites to generate NLCA and NLNTD, respectively. Sequences encoding a full-length CA of HIV-2 GL-AN (Pro1-Met231 in **Figure 1A**) and its NTD (Pro1-Tyr145 in **Figure 1A**) were PCR-amplified and cloned into pET21 as above to generate GLCA and GLNTD, respectively. All mutant clones analyzed in this study, designated NL/GL, GL32NLCA, and GLmtCA, were generated by overlapping PCR. Infectious molecular clones designated NL4-3 and GL-AN have been previously described (Adachi et al., 1986; Shibata et al., 1990; Kawamura et al., 1994). NL4-3 and GL-AN are prototype full-length clones of HIV-1 and HIV-2, respectively, and frequently and widely used for various HIV studies as representative clones.

### Expression and Purification of CA Proteins

**FIGURE 2:**
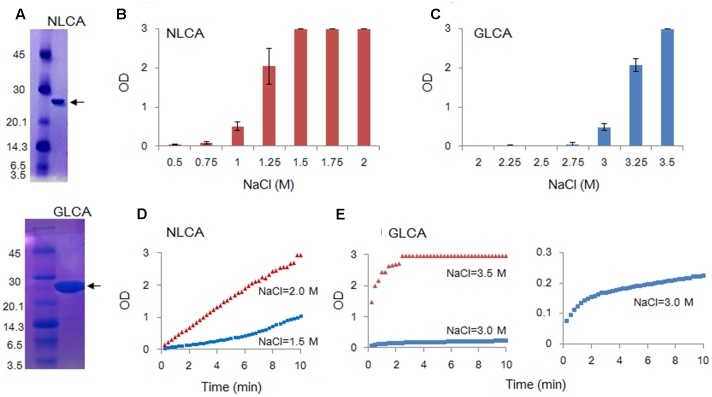
*In vitro* polymerization features of NL4-3 and GL-AN CA proteins. Polymerization reaction was carried out and monitored by OD at 350 nm as described in MATERIALS AND METHODS. **(A)** SDS-PAGE profile. The purity of CA proteins was checked by SDS-PAGE gel stained with Coomassie Brilliant Blue. Size markers in kDa (on the left) and the CA proteins (arrows) are indicted. NLCA, NL4-3 CA; GLCA, GL-AN CA. **(B**–**E)** Self-polymerization of CA proteins. Purified CA proteins were added to the buffer containing various concentrations of NaCl, and incubated for 4 h **(B–C)** or 10 min (**D–E)** at room temperature. Polymerized products and polymerization kinetics at various concentrations of NaCl were determined by OD at 350 nm for NLCA **(B,D)** and GLCA **(C,E)** as shown.

Recombinant CA proteins (tagged with poly-histidine at the C-terminus) were expressed in *E. coli* strain BL21 (DE3) RIL (Agilent technologies) and purified as previously described (Li et al., 2000). Expression of wild-type and mutant CA proteins were induced with 0.1 mM isopropyl β-D-thiogalactopyranoside. Recombinant proteins were then purified by immobilized metal affinity chromatography (TALON, Clontech Laboratories Inc.), and their purities were checked and confirmed by SDS-poly-acrylamide gel electrophoresis (SDS-PAGE) for subsequent experimental uses (**Figure 2A**).

### *In Vitro* Assay for HIV CA Assembly

Assays for CA assembly were performed similarly as described previously (Ehrlich et al., 1992; Li et al., 2000; Ganser-Pornillos et al., 2004; Barklis et al., 2009). Three independent polymerization experiments were carried out, and mean values ± standard deviations are shown where indicated. CA polymerization reactions were performed in 50 mM Tris-HCl (pH 8.0) at a final concentration of 50 μM recombinant CA proteins. Reactions were carried out at different NaCl concentrations for the indicated time in each experiment. Polymerized products were monitored by optical density (OD) at 350 nm using a spectrophotometer (DU730, Beckman–Coulter). OD_350_ has been used to most sensitively measure the ordered protein aggregates, not the protein itself. The upper OD detection limit of DU730 is 3.0.

### Transmission Electron Microscopy (TEM)

Negative staining and electron microscopy were performed similarly as described before (Sakaguchi et al., 2002; Piroozmand et al., 2006). NL4-3 and GL-AN CA proteins adjusted to 100 μM were *in vitro* polymerized in the presence of 2 M NaCl (for NL4-3) or 3.5 M NaCl (for GL-AN) for 30 min at room temperature. Assembled CA proteins were fixed by 0.2% glutaraldehyde, and were placed on formvar-carbon-coated nickel grids, stained with 4% uranyl acetate, and examined by a transmission electron microscope (Hitachi H-7650).

### Fluorescence-based Thermal Shift Assay

Fluorescence-based thermal shift assays by differential scanning fluorimetry (DSF) were carried out as described previously (Niesen et al., 2007; Fedorov et al., 2012). Three independent DSF experiments were conducted with highly similar results. Fifty μM of CA NTD proteins were prepared in 50 mM Tris-HCl (pH 8.0), 250–2000 mM NaCl and 1 mM 2-mercaptethanol containing SYPRO orange (Invitrogen) to quantify thermal stability. Temperature gradient was set in the range of 25°C to 95°C with 1% ramp rate using 7500 real-time PCR system (Applied Biosystems). Melting temperature (Tm) of CA was calculated by SYPRO orange fluorescence curves using 7500 software ver. 2.03.

## Results

### Polymerization Properties of HIV-1 and HIV-2 CA Proteins

Capsid protein is a major component of retroviral particles, and commonly plays an essential role for virus replication (Goff, 2013). Therefore, amino acid sequences in CA proteins and their structural features are expected to be conserved among viruses, especially those belonging to the same viral genus. Indeed, when HIV-1 and HIV-2 CA proteins are compared (**Figure 1**), their amino acid identities are around 70% (67% for NTD and 73% for CTD), and structural outlines of NTD proteins as revealed by nuclear magnetic resonance are highly similar (Price et al., 2009). Nevertheless, we were interested in ascertaining if there is some biochemical/biophysical difference(s) between the two closely related CA molecules from HIV-1 and HIV-2 that may potentially affect their biological properties.

**FIGURE 3:**
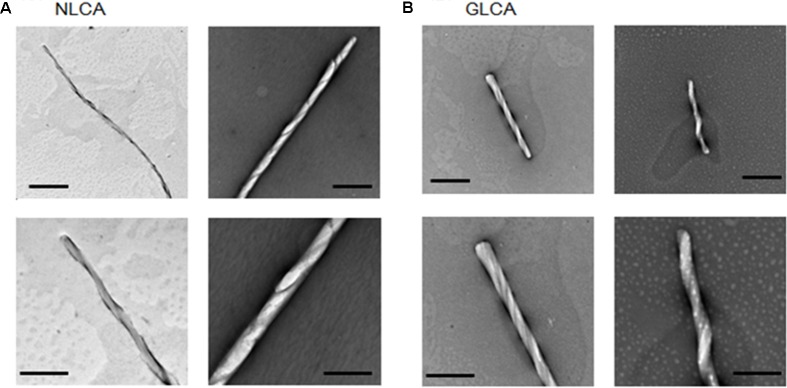
Tubular structures of *in vitro* assembled NL4-3 and GL-AN CA proteins. CA proteins were fully polymerized *in vitro*, fixed by glutaraldehyde, and visualized via TEM as previously described (Sakaguchi et al., 2002; Piroozmand et al., 2006). **(A,B)** represent micrographs of HIV-1 NL4-3 CA (NLCA) and HIV-2 GL-AN CA (GLCA), respectively. Scale bars: 1 μm in upper panels; 200 nm in lower panels.

To comparatively determine the physicochemical characteristics of HIV-1 and HIV-2 CA proteins, we employed the *in vitro* assembly system by NaCl (Ehrlich et al., 1992; Li et al., 2000; Ganser-Pornillos et al., 2004; Barklis et al., 2009) in this study. Recombinant CA proteins (derived from HIV-1 NL4-3 and HIV-2 GL-AN) expressed in bacteria and purified were used for monitoring their polymerization reactions. As shown in **Figures 2B,C**, a very prominent difference was noted between NL4-3 and GL-AN CA proteins for their NaCl-dependent polymerization. NL4-3 CA polymerized at a significant level with 1 M NaCl, and fully assembled with 1.5 M, 1.75 M, and 2 M NaCl. In a sharp contrast, no polymerized products were detected for GL-AN CA with 2.75 M and lower concentrations of NaCl. At least 3 M NaCl or higher concentrations were required for GL-AN CA to definitively detect the polymerization products. We then performed kinetic studies to determine whether there was any difference between NL4-3 and GL-AN CA proteins (**Figures 2D,E**). As demonstrated, NL4-3 CA self-assembled in a linear way over time with 1.5 M and 2.0 M NaCl, whereas GL-AN CA exhibited Boltzmann shape curves with 3 M and 3.5 M NaCl even when OD values were very low (note the GLCA right panel in **Figure 2E**). In total, GL-AN CA self-polymerized more rapidly than NL4-3 CA once successfully triggered by NaCl. Considering early reports on the TEM morphology of *in vitro* HIV-1 CA products (Ehrlich et al., 1992; Campbell and Vogt, 1995; Gross et al., 1997), we checked for the presence of assembled CA proteins with a tubular or cylinder shape in the reaction products. As shown in **Figures 3A,B**, polymers with a similar morphology were readily observed in the *in vitro* assembled products of NL4-3 and GL-AN CA proteins, as previously described.

### Polymerization Properties of CA Chimeric and Mutant Proteins

**FIGURE 4:**
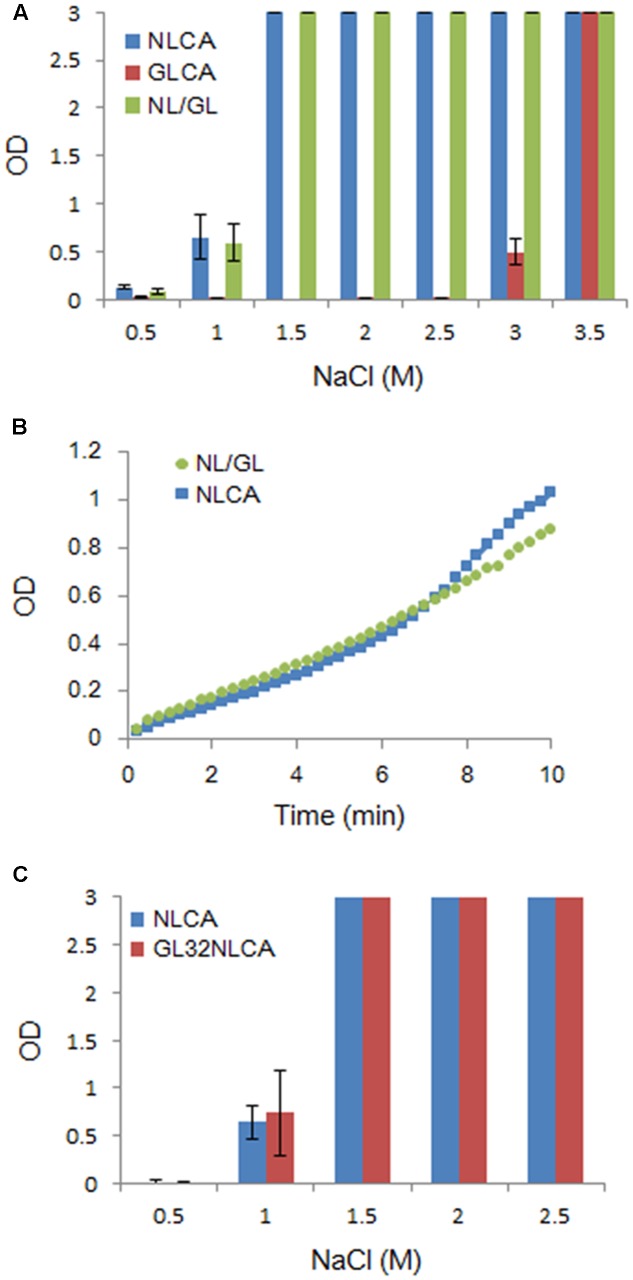
Comparative analysis of several CA proteins for their *in vitro* polymerization properties. CA-polymerization was performed *in vitro* and monitored by OD at 350 nm as described in MATERIALS AND METHODS. **(A)** Polymerization of NL4-3 CA (NLCA), GL-AN CA (GLCA), and NL/GL CA (NL/GL) proteins for 4 h at various NaCl concentrations. The chimeric clone NL/GL has the sequence encoding the NTD of NL4-3 CA and the linker domain/CTD of GL-AN CA (**Figure 1A**). **(B)** Polymerization kinetics of NL and NL/GL CA proteins (1.5 M NaCl). **(C)** Polymerization of NL and GL32NL CA proteins for 4 h at various NaCl concentrations. GL32NL is a chimeric NLCA-derivative clone which has the sequence encoding the very N-terminal region of GL-AN CA (Pro1-Phe32 in **Figure 1A**).

Although the amino acid identities in NL4-3 and GL-AN CA proteins are considerably high (**Figure 1**), their polymerization properties *in vitro* were clearly distinct (**Figure 2**). To determine the region in CA responsible for the observed differences, we first constructed a chimeric clone between the two CA proteins and designated it as NL/GL. NL/GL contains the NTD of NL4-3 CA and the linker/CTD of GL-AN CA (**Figure 1A**). We then performed the *in vitro* polymerization assays for NL4-3 CA, GL-AN CA, and NL/GL as described above. As clearly seen in **Figure 4A**, NL/GL gave very similar results with those obtained for NL4-3 CA, but very different from those for GL-AN CA. Consistent with these results, NL/GL polymerization kinetics were highly similar to those of NL4-3 CA (**Figure 4B**). We concluded that NTD determines differences in *in vitro* polymerization properties of NL4-3 and GL-AN CA proteins.

It has been reported that the N-terminal β-hairpin structure is important for CA assembly by analyzing a chimeric protein between HIV-1 and murine leukemia virus CA proteins (Cortines et al., 2011). This suggested that the most N-terminal region containing the β-hairpin structure could be determinants for the different polymerization phenotypes between NL4-3 and GL-AN CA proteins. We therefore constructed a chimeric CA clone designated GL32NLCA to test this possibility. The N-terminal portion of NLCA (Pro1-Phe32 in **Figure 1A**) was replaced with the corresponding region of GLCA to generate GL32NLCA. As shown in **Figure 4C**, when NLCA and GL32NLCA were monitored for the polymerization activity at various NaCl concentrations, no significant difference was noted. This result indicated that the region containing the N-terminal β-hairpin structure is not a determinant for the distinct polymerization properties.

**FIGURE 5:**
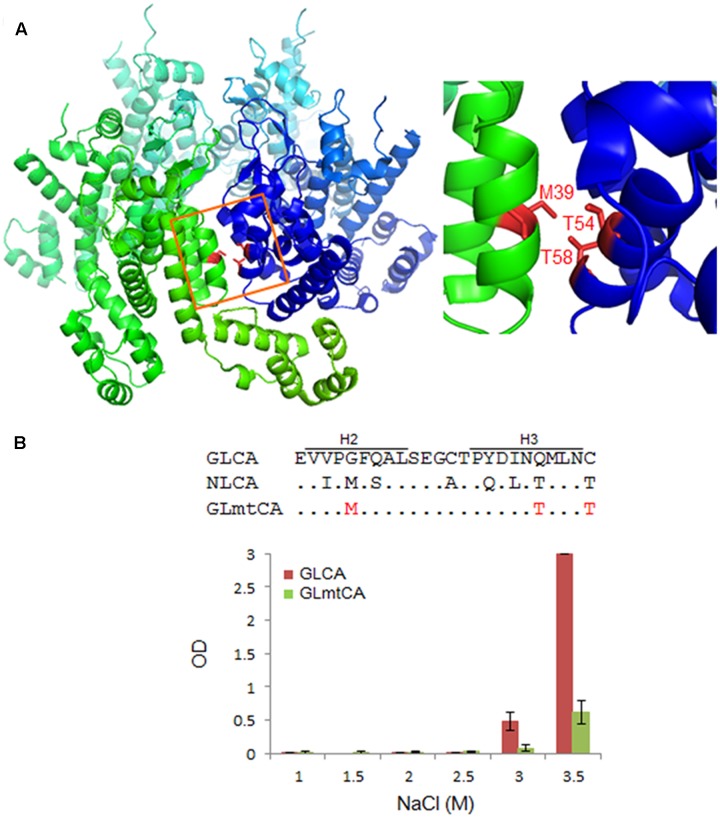
Comparison of *in vitro* polymerization activity of GL-AN and mutant CA proteins. **(A)** CA–CA interacting surface of HIV-1. Two CA molecules (greenish and bluish) are shown. Three amino acids (M39, T54, and T58 in the H2–H3 region) critical for the interaction are highlighted as shown (see Pornillos et al., 2009, 2011 for details). The structure of HIV-1 CA (PDB code: 3H4E) was depicted by PyMOL ver 1.8. **(B)** Polymerization of parental and mutant CA proteins. CA-polymerization was performed *in vitro* for 4 h at various NaCl concentrations, and monitored by OD at 350 nm as described in the Section “Materials and Methods”. The mutant clone GLmtCA has three amino acid substitutions relative to GLCA as indicated.

Previous structural studies have shown that some amino acid residues (no. 20, 38, 39, 42, 54, and 58 in CA) located at the CA–CA interaction interface (regions of helices 1, 2, and 3) are critical for polymerization (Pornillos et al., 2009, 2011). Of these six residues, three, i.e., L20, P38, and A42, are conserved between NL4-3 and GL-AN CA proteins (**Figure 1A**). Consequently, three amino acids (no. 39, 54, and 58) are unshared, and reside in the CA–CA interacting surface (**Figure 5**). We therefore introduced three amino acid substitutions (G39M, Q54T, and C58T) into GLCA to generate GLmtCA carrying residues of the NL4-3 CA type (**Figure 5**), and examined its polymerization ability. GLmtCA was found to have much less polymerization activity than GLCA (**Figure 5B**). This result indicated that amino acids G39, Q54, and C58 are critical for self-assembly of GL-AN CA, and that the amino acid substitutions introduced cannot change its polymerization property to the NL4-3 type.

### Thermal Stability of CA NTD Proteins

It has been reported that chemical chaperons, inhibitors of HIV-1 CA polymerization, raise its Tm (Lampel et al., 2013). Furthermore, previous studies have reported that higher temperature facilitates the polymerization of HIV-1 CA (Ehrlich et al., 1992; Morikawa et al., 2004; Alfadhli et al., 2005). Moreover, the interaction of HIV CA with CypA or anti-retroviral TRIM-Cyp, known to promote HIV CA dissociation (Braaten et al., 1996a,b; Gamble et al., 1996; Forshey et al., 2005; Sebastian and Luban, 2005; Stremlau et al., 2006), was shown to be an exothermic event (Yoo et al., 1997; Price et al., 2009), indicating that HIV CA is shifted to a more thermally stable state upon binding to CypA or TRIM-Cyp. These results have strongly suggested that the ability of CA to polymerize and the thermal stability of CA are mutually linked. We therefore comparatively determined the thermal stability of CA NTD proteins derived from NL4-3 and GL-AN, the determinants for distinct polymerization properties of the two CA proteins (**Figure 4**).

**FIGURE 6:**
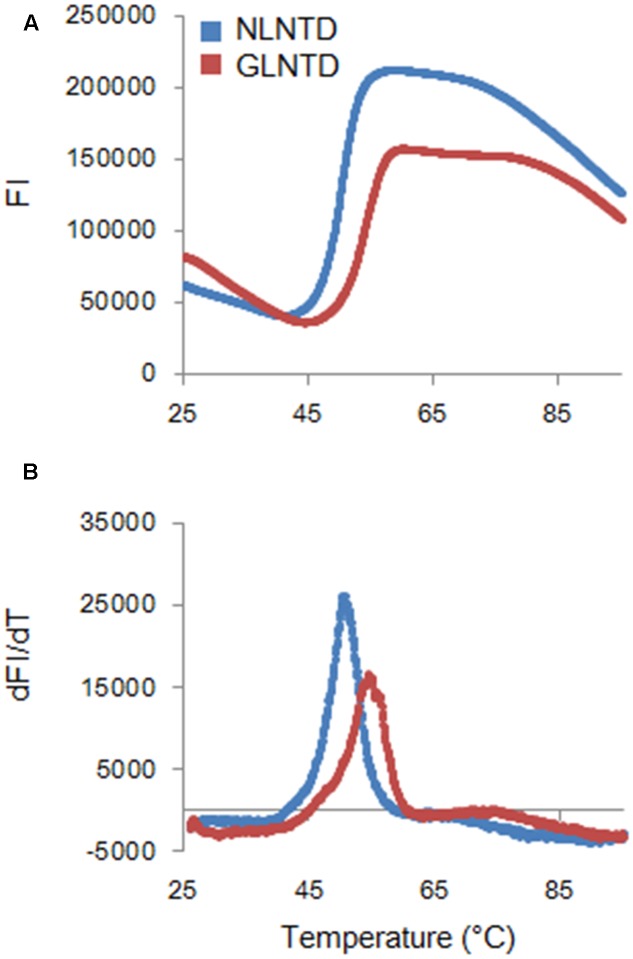
Thermal stability of CA NTD proteins derived from HIV-1 NL4-3, and HIV-2 GL-AN. The thermal stability of NLCA and GLCA NTD proteins in the presence of 250 mM NaCl was determined by DSF as described in the Section “Materials and Methods”. SYPRO orange fluorescence intensity (FI) at varying temperatures (upper panel) and derivative melt curves calculated by differences in FI at each temperature (lower panel) are shown. Peak temperatures in the curves (dFI/dT) were considered as Tm.

**FIGURE 7:**
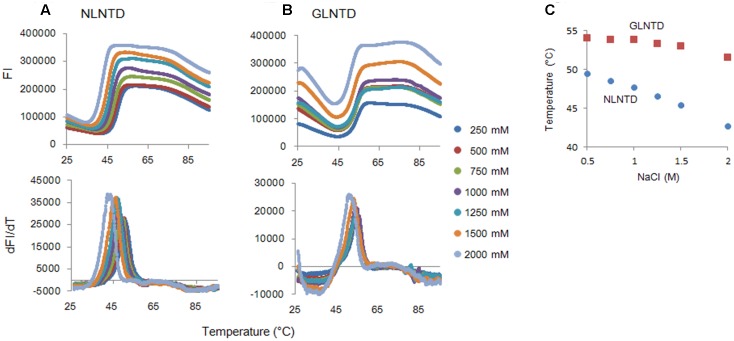
Thermal stability of CA NTD proteins at various NaCl concentrations. The thermal stability of NLCA and GLCA NTD proteins at various NaCl concentrations was evaluated as described in the legend to **Figure 6**. FI values and melt curves (**A,B**, respectively), and Tm shifts induced by NaCl **(C)** are shown.

To determine the Tm for the two proteins, we employed fluorescence-based thermal shift assays using SYPRO orange. Proteins expose hydrophobic patches upon heating. This assay utilizes a chemical property of SYPRO orange to bind to hydrophobic patches in target proteins, and therefore, their denaturation states can be monitored by fluorescence intensities from SYPRO orange. As shown in **Figure 6**, fluorescence intensity curves of NL4-3 and GL-AN NTD proteins obtained by this assay system were quite different, and Tm values for NL4-3 NTD and GL-AN NTD were calculated to be 50.4 °C and 53.9 °C, respectively. Thus, GL-AN NTD was thermally more stable than NL4-3 NTD. We further examined the effects of NaCl, an agent to promote CA polymerization (**Figure 2**), on the thermal stability of the two NTD proteins. **Figure 7** shows the results obtained. The Tm for NL4-3 NTD fell down (7.4 °C) by increasing NaCl concentrations from 250 mM to 2000 mM (**Figures 7A,C**). On the other hand, relatively mild effects were observed for the Tm-shift of GL-AN NTD (2.1°C falling down as shown in **Figures 7B,C**). Thus, GL-AN NTD was less influenced by NaCl with respect to thermal stability than NL4-3 NTD. In other words, NL4-3 NTD was structurally destabilized to a greater extent by NaCl than GL-AN NTD. Collectively, GL-AN NTD was structurally more stable than NL4-3 NTD.

## Discussion

Although there have been numerous studies on the assembly of HIV-1 CA *in vitro* (Ehrlich et al., 1992, 2001; Campbell and Vogt, 1995; Gross et al., 1997, 2000; von Schwedler et al., 1998; Lanman et al., 2002; Mateu, 2002;Morikawa et al., 2004; Alfadhli et al., 2005; del Alamo et al., 2005; Lidon-Moya et al., 2005; Chen and Tycko, 2010), almost no experimental investigations into the corresponding research field of HIV-2 have been done as of yet. In this work, we determined *in vitro* characteristics of HIV-2 CA (derived from an infectious clone, GL-AN) in parallel with HIV-1 CA (derived from an infectious clone, NL4-3). We confirmed previous findings on HIV-1 CA, and newly found that HIV-2 CA is strikingly distinct from HIV-1 CA regarding its *in vitro* properties (**Figures 2**, **4**, **6**, **7**) despite their sequence relatedness and structural similarity (**Figure 1**). We demonstrated here that much higher concentrations of NaCl are required for the polymerization of HIV-2 CA than for HIV-1 CA, but that HIV-2 CA assembly proceeds more promptly relative to HIV-1 CA after being initiated (**Figures 2**, **4**). Although a specific narrow region in CA was not identified as a determinant, NTD is clearly responsible for this property (**Figure 4**). This conclusion is quite reasonable because amino acid identities are higher in CTD than those in NTD, and would be consistent with the finding that intermolecular CTD-CTD interaction occurs first and NTD-NTD interaction occurs as the final step (Grime and Voth, 2012; Tsiang et al., 2012). NTD may function as a rate-limiting factor for CA polymerization.

We also demonstrated by fluorescence-based thermal shift assays that HIV-2 CA NTD is structurally more stable than HIV-1 CA NTD (**Figures 6**, **7**). Thus, the thermal stability of NTD proteins was inversely related with the polymerization ability of CA proteins at lower NaCl concentrations (**Figures 2**, **4**). Although the molecular basis is still unknown, the negative relationship between thermal stability and assembly property has been reported for CA proteins of HIV-1 and its CA mutant (Ganser-Pornillos et al., 2004; Cortines et al., 2015), and for HIV-1 CA treated with chemical chaperons (Lampel et al., 2013). Furthermore, HIV-1 CA polymerization was facilitated by heat destabilization (Ehrlich et al., 1992; Morikawa et al., 2004; Alfadhli et al., 2005; Barklis et al., 2009). Taken together, it is not unreasonable to assume that the relatively high thermal stability of CA is associated with the relatively poor assembly property (i.e., high disassembly property) of CA. However, whether the different sequence and/or structural property of HIV-1/HIV-2 CA proteins needed to bind to cellular restriction or regulation factors, such as TRIM5α or CypA, are linked with our results described here is unclear at present. The molecular basis underlying the distinct *in vitro* features of HIV-1/HIV-2 CA proteins remains to be elucidated.

HIV Gag-CA proteins play indispensable roles at various steps in the viral replication cycle (Freed and Martin, 2013). Our results described here clearly indicated that CA proteins of HIV-1 and HIV-2 are biochemically distinct. The data on the thermal stability of HIV-1/HIV-2 CA NTD proteins (**Figures 6**, **7**) may account for the unique polymerization properties of HIV-1 and HIV-2 CA proteins (**Figures 2**, **4**). As for the biological implications of our findings, we noticed a report showing that the HIV-2 GH123 virus carrying an identical Gag-CA with GL-AN exhibits faster uncoating (CA disassembly) kinetics in infected cells relative to the HIV-1 NL4-3 virus (Takeda et al., 2015). This observation is in good agreement with our results, supporting the notion that the instability/stability of CA proteins may affect the early viral replication phase (uncoating) of HIV-1 and HIV-2. The plausibility of our concept needs to be biochemically and biologically verified.

It is reasonable to consider Gag-CA as a therapeutic target, and indeed, there have been numerous attempts in this direction (Tang et al., 2003; Zhang et al., 2008; Tian et al., 2009; Blair et al., 2010; Jin et al., 2010; Curreli et al., 2011; Shi et al., 2011; Kortagere et al., 2012; Lemke et al., 2012; Lamorte et al., 2013; Matreyek et al., 2013; Bhattacharya et al., 2014; Fricke et al., 2014; Peng et al., 2014; Price et al., 2014). However, to the best of our knowledge, none of the anti-viral inhibitors described have proceeded to the steps to study their practical/clinical use. In the present study, we have demonstrated the close association of CA polymerization property *in vitro* and thermal stability of CA NTD as monitored by DSF. Thermal stability can be readily evaluated in large numbers simultaneously by a real-time PCR machine. It is thus practical to identify compounds by DSF that unusually destabilize or stabilize Gag-CA NTD proteins of HIV-1/HIV-2. In fact, a small molecule named PF74, previously reported to bind to HIV-1 CA and induce premature HIV-1 uncoating (Shi et al., 2011), was found to aberrantly increase the stability of NLCA NTD as revealed by DSF in our pilot experiments (manuscript in preparation). The system based on DSF (**Figures 6**, **7**) represents a promising new high-throughput screening method to search for durable and effective anti-HIV CA antivirals from a large library of candidate molecules.

## Author Contributions

YS, AA, and MN designed the research project. YS, AM, and TU performed the experiments. YS, AM, ND, TK, TU, AA, and MN discussed the results obtained. YS, TK, AA, and MN wrote the manuscript. All authors approved its submission.

## Conflict of Interest Statement

The authors declare that the research was conducted in the absence of any commercial or financial relationships that could be construed as a potential conflict of interest.
